# Reinvestigation into the role of lipopolysaccharide Glycosyltransferases in *Helicobacter pylori* protein glycosylation

**DOI:** 10.1080/19490976.2025.2455513

**Published:** 2025-01-20

**Authors:** Hong Li, Xiaoqiong Tang, Tiandi Yang, Tingting Liao, Aleksandra W. Debowski, Tiankuo Yang, Yalin Shen, Hans-Olof Nilsson, Stuart M. Haslam, Barbara Mulloy, Anne Dell, Keith A. Stubbs, Wolfgang Fischer, Rainer Haas, Hong Tang, Barry J. Marshall, Mohammed Benghezal

**Affiliations:** a Center of Infectious Diseases, West China Hospital of Sichuan University, Chengdu, China; b Laboratory of Infectious and Liver Diseases, Institute of Infectious Diseases, West China Hospital of Sichuan University, Chengdu, China; c Helicobacter pylori Research Laboratory, Marshall Centre for Infectious Disease Research and Training, School of Biomedical Sciences, University of Western Australia, Nedlands, Australia; d Department of Life Sciences, Imperial College London, London, UK; e School of Molecular Sciences, University of Western Australia, Crawley, Australia; f Max von Pettenkofer Institute of Hygiene and Medical Microbiology, Faculty of Medicine, and German Center for Infection Research (DZIF), LMU Munich, Munich, Germany; g Medical Microbiology and Hospital Epidemiology, Max von Pettenkofer Institute, Faculty of Medicine, LMU Munich, Munich, Germany

**Keywords:** *Helicobacter pylori*, molecular weight shift, SDS-PAGE, protein glycosylation, lipopolysaccharide

## Abstract

Protein glycosylation has been considered as a fundamental phenomenon shared by all domains of life. In *Helicobacter pylori*, glycosylation of flagellins A and B with pseudaminic acid have been rigorously confirmed and shown to be essential for flagella assembly and bacterial colonization. In addition to flagellins, several other proteins including RecA, AlpA/B, and BabA/B in *H. pylori* have also been reported to be glycosylated and to be dependent on the lipopolysaccharide (LPS) biosynthetic pathway. However, these proteins have not been purified for sugar-specific staining or structural analysis to confirm the existence of carbohydrate motifs. Here, using a combined approach of genetics, protein purification, and sugar-specific staining, we demonstrate that RecA is not a glycoprotein. Moreover, using LPS-protein reconstitution experiments, we demonstrate that the presence of O-antigen containing full-length LPS interferes with the electrophoretic mobility of *H. pylori* RecA and many other proteins including AlpA/B on SDS-PAGE. Finally, we demonstrate that full-length LPS extracted from *E. coli* affects electrophoretic migration of *H. pylori* proteins, while full-length LPS extracted from *H. pylori* similarly influences the electrophoretic migration of *E. coli* proteins. The impact is more subtle with *E. coli* LPS compared to *H. pylori* LPS, indicating that the magnitude of effect of LPS effects on protein mobility is dependent on bacterial source of the LPS. These findings suggest that the effects of full-length LPS on protein electrophoresis may represent a more general phenomenon. As LPS is a unique component of virtually all Gram-negative bacteria, our data suggest that when observing protein electrophoretic mobility shifts between wild-type and LPS mutant strains or between subcellular fractionation samples, the influence of LPS on protein electrophoretic migration should be considered first, rather than interpreting it as potential protein glycosylation that is dependent upon LPS biosynthetic pathway.

## Introduction

The human gastric pathogen *Helicobacter pylori* is a Gram-negative and spiral-shaped bacterium, infecting more than 40% of the world’s population.^1–3^ As *H. pylori* infection is always transmissible, *H. pylori* gastritis has been defined as an infectious disease in all recent guidelines for *H. pylori* management.^4–6^ Although mostly asymptomatic, virtually all *H. pylori*-infected subjects have chronic active gastritis of varying severity,^5^ which may develop into peptic ulcers, atrophic gastritis, mucosa-associated lymphoid tissue (MALT) lymphoma, and gastric cancer.

Protein glycosylation is considered a fundamental phenomenon shared by all domains of life.^7^ Over the last decade, several proteins in *H. pylori* have been reported to be glycosylated including flagellin proteins FlaA and FlaB,^8,9^ RecA,^10^ AlpA/B,^11,12^ and BabA/B.^12^ To date, however, only FlaA and FlaB have been rigorously confirmed to be glycoproteins using a combination of genetic, biochemical, structural, and functional approaches.^11,13^ Flagellins are glycosylated with pseudaminic acid (Pse) by the glycosyltransferase PseE (HP0114) using the glycosyl donor CMP-Pse^8,14^ and six Pse biosynthesis enzymes (PseB, C, H, G, I, F) have been found to constitute the complete CMP-Pse biosynthetic pathway starting from UDP-GlcNAc.^8,15^ *H. pylori* flagellin glycosylation is essential for flagella assembly, bacterial motility, and the colonization of the host stomach.^15^

RecA,^10^ AlpA/B,^11,12^ and BabA/B^12^ in *H. pylori* have been proposed to be post-translationally modified by glycosylation using glycosyltransferases involved in LPS biosynthesis. In the specific case of RecA, mutation of two LPS biosynthetic genes *galE* (*HP0360*, involved in the generation of UDP-Gal), and *pmi* (*HP0043*, involved in the generation of GDP-L-Fuc) resulted in an increase of electrophoretic mobility of RecA on SDS-PAGE gel.^10^ The decreased molecular weight (MW) of RecA deduced from the increased electrophoretic mobility was interpreted as the loss of RecA glycosylation upon mutation in LPS biosynthetic genes.^10^ Likewise, the increased electrophoretic migration of AlpA/B and BabA/B upon mutation of the LPS O-antigen biosynthetic genes encoding WecA, Wzk, and WaaL in *H. pylori* was interpreted as the loss of protein glycosylation.^12^ In addition, the adhesin protein EmaA in *Aggregatibacter actinomycetemcomitans* was also found to exhibit an increased electrophoretic mobility upon the mutation of three LPS biosynthetic enzymes RmlC (a rhamnose sugar biosynthetic enzyme), the O-antigen flippase (Wzt), and the O-antigen ligase (WaaL).^16^ Therefore, it has been proposed that AlpA/B and BabA/B in *H. pylori*, and EmaA in *A. actinomycetemcomitans* are all glycosylated in a LPS biosynthetic machinery-dependent manner.^16^ However, no additional biochemical or structural data are available to confirm the existence of glycan decoration of RecA, AlpA/B, and BabA/B in *H. pylori*, nor the glycan decoration of EmaA in *A. actinomycetemcomitans*. Therefore, a definitive conclusion that *H. pylori* employs a general protein glycosylation system that is dependent on LPS biosynthesis remains elusive.

In this study, using a combined approach of genetics, protein purification, sugar-specific staining, LPS structural analysis, and LPS-protein reconstitution experiments, we demonstrate that RecA is not a glycoprotein, and that the presence of full-length LPS alters the electrophoretic mobility of RecA and many other proteins, whereas O-antigen deficient or truncated LPS does not alter protein migration on SDS-PAGE. As LPS is a unique component of virtually all Gram-negative bacteria, our data suggest that when deciphering an observed decrease in protein MW on SDS-PAGE upon disruption of the LPS biosynthetic pathway in Gram-negative bacteria, the influence of the resultant different sizes of LPS on SDS-PAGE protein migration should be considered first, rather than interpreting the MW shift as evidence of potential protein glycosylation dependent on LPS biosynthetic machinery.

## Results

### LPS of different length and structure correlates with the RecA protein electrophoretic shift

**Figure 1.**
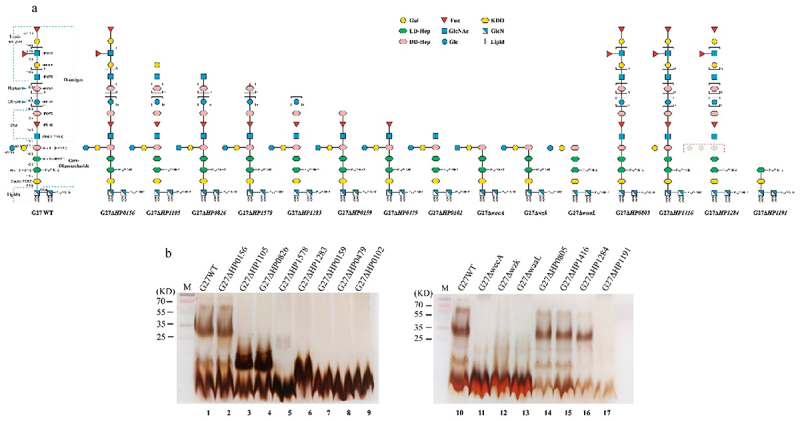
LPS structures of G27 wild-type and 15 mutants. (a) Schematic of LPS structures from G27 wild-type and 15 mutant strains used in this study. (b) LPS profiles of G27 wild-type and mutants separated on 15% SDS-PAGE and visualized by silver staining. Gal, galactose; Fuc, fucose; KDO, 3-deoxy-D-mannose-octanoic acid; Hep, heptose; GlcNAc, *N*-acetylglucosamine; GlcN, glucosamine.

To investigate the association of the apparent RecA MW shift with LPS biosynthesis, *H. pylori* wild-type strain G27 and a panel of 15 isogenic mutants were included in this study (Figure 1, Table S1).^13^ Based on biochemical and structural analyses of G27 wild-type and these mutants, we have previously redefined the *H. pylori* G27 LPS core-oligosaccharide as a conserved short hexasaccharide (Glc-Gal-DD-Hep-III-LD-Hep-II-LD-Hep-I-KDO) attached to a long O-antigen consisting of a conserved trisaccharide (Trio, DD-Hep-Fuc-GlcNAc), a glucan, a heptan, and the terminal Lewis antigen).^17,18^ Notably, *HP0156* is not a glycosyltransferase gene; however, the mutation of this gene in *H. pylori* strain P1 was reported to result in a decrease in the apparent MW of RecA compared with the parent strain.^10^ Therefore, the G27Δ*HP0156* mutant was constructed in this study, and its LPS structure was further analyzed using mass spectrometry (MS)-based strategies as in previous studies.^17,18^ The MS data indicated that the general G27Δ*HP0156* LPS structure is similar to G27 wild-type LPS (Figure 1 and S1). The schematic LPS structures of G27 wild-type and the 15 mutants are summarized in Figure 1a, and their LPS profiles were compared by silver staining (Figure 1b).

**Figure 2.**
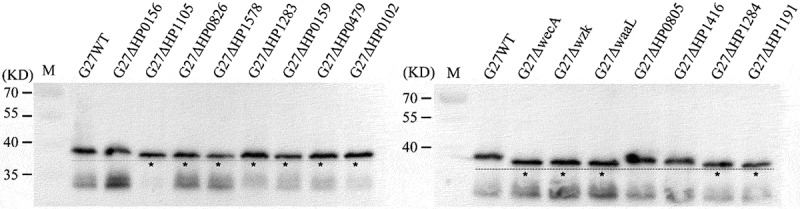
The apparent MW of RecA in G27 wild-type and 15 mutants. Whole cell lysates were analyzed by Western blot using an anti-RecA antibody. Samples were prepared from equal amounts of bacteria (standardized by OD600). Lanes in which the apparent MW of RecA appears to be smaller than that of wild-type are indicated by an asterisk.

We next investigated the electrophoretic mobility of RecA from G27 wild-type and the 15 mutants. Interestingly, compared to the MW of wild-type RecA, the MW of RecA was observed to be smaller in 12 mutants that expressed truncated or abnormal LPS (Figure 2). These mutants include G27Δ*HP1105*, G27Δ*HP0826*, and G27Δ*HP1578*, all of which exhibited truncation of the terminal Lewis antigen; G27Δ*HP1283*, where the LPS is truncated after the glucan; G27Δ*HP0159*, where the LPS is truncated after the conserved Trio moiety; G27Δ*HP0479* and G27Δ*HP0102*, where the LPS is truncated after the Fuc and Glc residues of the Trio, respectively; G27Δ*wecA*, G27Δ*wzk*, and G27Δ*waaL*, where the LPS is completely devoid of the O-antigen; G27Δ*HP1191*, where the LPS is truncated after Hep-I residue; and G27Δ*HP1284*, which lacks the core Hep-III and the adjoining Glc-Gal residues but still retains the O-antigen attached through the Hep-II residue. However, for the remaining 3 mutants (G27Δ*HP0156*, G27Δ*HP0805*, and G27Δ*HP1416*) which display a full-length LPS, the apparent MW of RecA was comparable to that of RecA from G27 wild-type (Figure 2).

This data demonstrates a clear correlation between LPS length and structure, and RecA protein electrophoretic shift. The observed decrease in the apparent MW of RecA in the 12 LPS mutants harboring abnormal core or truncated LPS length led us to suspect that the interpretation of a shift in apparent MW of RecA as being protein glycosylation-mediated and dependent on LPS biosynthesis is unlikely. In addition, if all the corresponding enzymes involved in LPS biosynthesis are shared with RecA glycosylation, the protein should be modified with the whole O-antigen and have a MW far more than the observed 2 kDa shift in apparent MW.^17,18^

### RecA is not acted upon by LPS biosynthetic machinery

**Figure 3.**
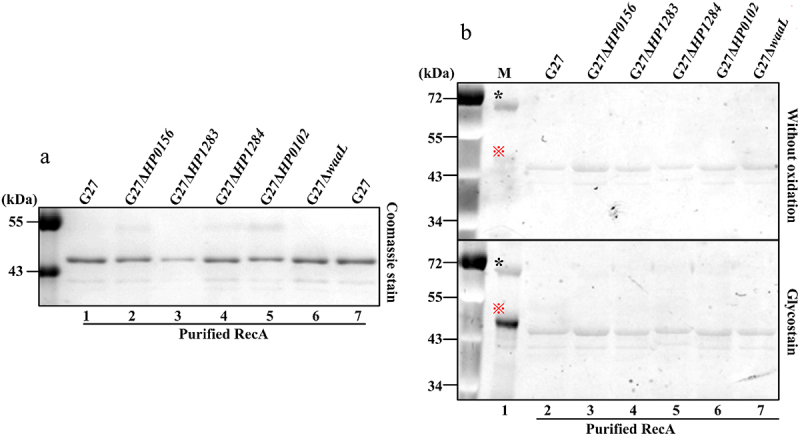
Glycol-staining of purified RecA. (a) Coomassie stain of the purified His6-tagged RecA from G27 wild-type and mutants. (b) Glycol staining of the purified His6-tagged RecA. The upper and the lower gels were the same samples, and the oxidation step was omitted in the upper gel as a control. Lane 1: glycol staining control (*unglycosylated albumin, 66 kDa, negative control; ※*N*-linked glycoprotein ovalbumin, 45 kDa, positive control); lanes 2–7: the His6-tagged RecA protein purified from G27 wild-type and mutant strains.

To analyze whether glycosylation is responsible for the observed electrophoretic shift of RecA, a His_6_-tagged RecA was expressed in G27 wild-type and five selected mutants (G27Δ*HP1283*, G27Δ*HP1284*, G27Δ*HP0102*, G27Δ*waaL*, and G27Δ*HP0156*) and purified. We reasoned that if the LPS biosynthetic machinery is indeed involved in glycosylation of RecA, the MW of His_6_-tagged RecA purified from G27Δ*HP0156* and G27 wild-type would be higher than the His_6_-tagged RecA purified from the other four mutants which synthesized truncated LPS (G27Δ*HP1283*, G27Δ*HP1284*, G27Δ*HP0102*, and G27Δ*waaL* RecA). However, RecA purified from all six strains exhibited the same apparent MW on Coomassie-stained SDS-PAGE (Figure 3a). In addition, none of the purified RecA exhibited staining indicative of the presence of glycosylation (Figure 3b). Thus, the loss of RecA electrophoretic shift after purification and the negative result for glycosylation suggests that RecA is not a glycoprotein.

### The presence of full-length H. pylori LPS alters the electrophoretic mobility of RecA and AlpA/B

**Figure 4.**
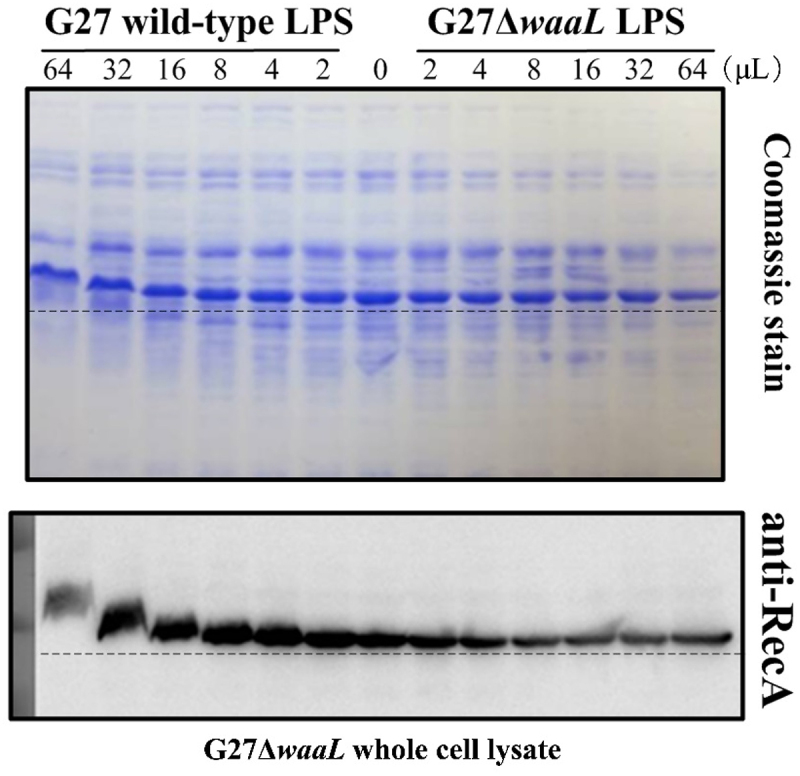
Up-titration of purified full-length LPS from G27 wild-type and O-antigen truncated LPS from G27Δ*waaL* into the whole cell lysate of G27Δ*waaL* for comparison of RecA electrophoretic mobility. The LPS up-titration was set up according to Table S3.

Previous subcellular fractionation experiments have shown that RecA from cytoplasmic fractions of wild-type and LPS mutants exhibited the same MW on SDS-PAGE, whereas RecA from membrane fractions of wild-type and mutants exhibited a shift in MW.^10^ Similarly, through subcellular fractionation, *H. pylori* adhesins including AlpA, AlpB, BabA, and BabB from 26,695 wild-type displayed an increase in apparent MW between the cytoplasm and the membrane fractions.^12^ We reasoned that the presence of full-length LPS in the membrane fraction is what affects protein electrophoretic mobility on SDS-PAGE, whereas LPS truncated of O-antigen has no influence on protein electrophoresis. To confirm this, we performed up-titration of purified full-length LPS from G27 wild-type and O-antigen truncated LPS from G27Δ*waaL* into the whole cell lysate of G27Δ*waaL* for comparison of RecA electrophoretic mobility. Remarkably, up-titration of full-length LPS from G27 wild-type resulted in a progressive increase of RecA apparent MW as observed on SDS-PAGE (Figure 4). In contrast, the apparent MW of RecA remained unchanged upon up-titration of purified LPS from the O-antigen mutant G27Δ*waaL* (Figure 4). This clearly indicates that the presence of full-length LPS alters RecA electrophoretic motility on SDS-PAGE, which can be easily misinterpreted as protein glycosylation.

**Figure 5.**
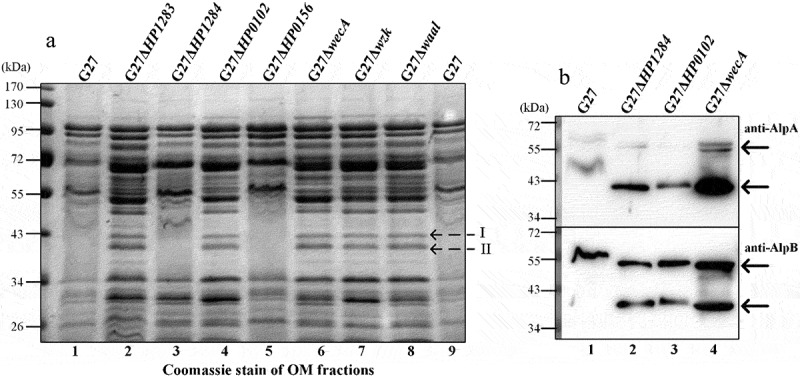
SDS-PAGE analysis of the outer membrane fraction protein profiles in G27 wild-type and mutant strains. (a) Coomassie stain of the outer membrane fractions of G27 wild-type and mutants (the denoted bands I and II were excised for MS analysis) (b) the outer membrane fractions of G27 wild-type and three mutant strains were probed with anti-AlpA and anti-AlpB (the two forms of AlpA/B are indicated by arrows). OM fractions were standardized by protein concentration, and the extracted samples were prepared from equal amounts of bacteria (standardized by OD600).

To further analyze the influence of the different size of LPS on *H. pylori* outer membrane proteins, we prepared the outer membrane fractions of *H. pylori* G27 wild-type and selected LPS mutants to compare the electrophoresis pattern of outer membrane proteins. Interestingly, an irregular electrophoresis pattern with V-shaped or diffused bands on Coomassie-stained SDS-PAGE gel was observed in the O-antigen containing strains (G27 wild-type, G27Δ*HP1284*, and G27Δ*HP0156*, Figure 5a, lanes 1, 3, 5, and 9), whereas a normal electrophoresis pattern with clear bands of outer membrane proteins was observed in all LPS O-antigen truncated mutants (Figure 5a, lanes 2, 4, and 6–8). This result indicates that O-antigen containing full-length LPS substantially alters the electrophoretic pattern of *H. pylori* outer membrane proteins.

Of note, 2 protein bands (denoted as I and II in Figure 5a) clearly present in sample preparations from the O-antigen truncated mutants appeared to be fuzzy or missing in samples from the O-antigen containing strains. The protein bands I and II were excised from the gel for identification by mass spectrometry. Outer membrane protein AlpA was identified in band I, and proteins HopA, AlpB, HPG27_119, and HPG27_1138 were identified in band II. The outer membrane fractions from G27 wild-type, mutants G27Δ*HP1284*, G27Δ*HP0102*, and G27Δ*wecA* were further subjected to Western blot analysis with AlpA- and AlpB-specific antibodies. As AlpA/B are heat-modifiable porins,^19,20^ two forms of AlpA and AlpB were detected by Western blot, and a clear shift in the apparent MW of AlpA/B was observed between samples from the wild-type and LPS mutants (Figure 5b).

Taken together, our results suggest that the presence of full-length LPS alters the electrophoretic mobility of RecA, AlpA/B and likely many other outer membrane proteins on SDS-PAGE.

### Alteration of protein electrophoretic mobility by LPS is not unique in H. pylori

**Figure 6.**
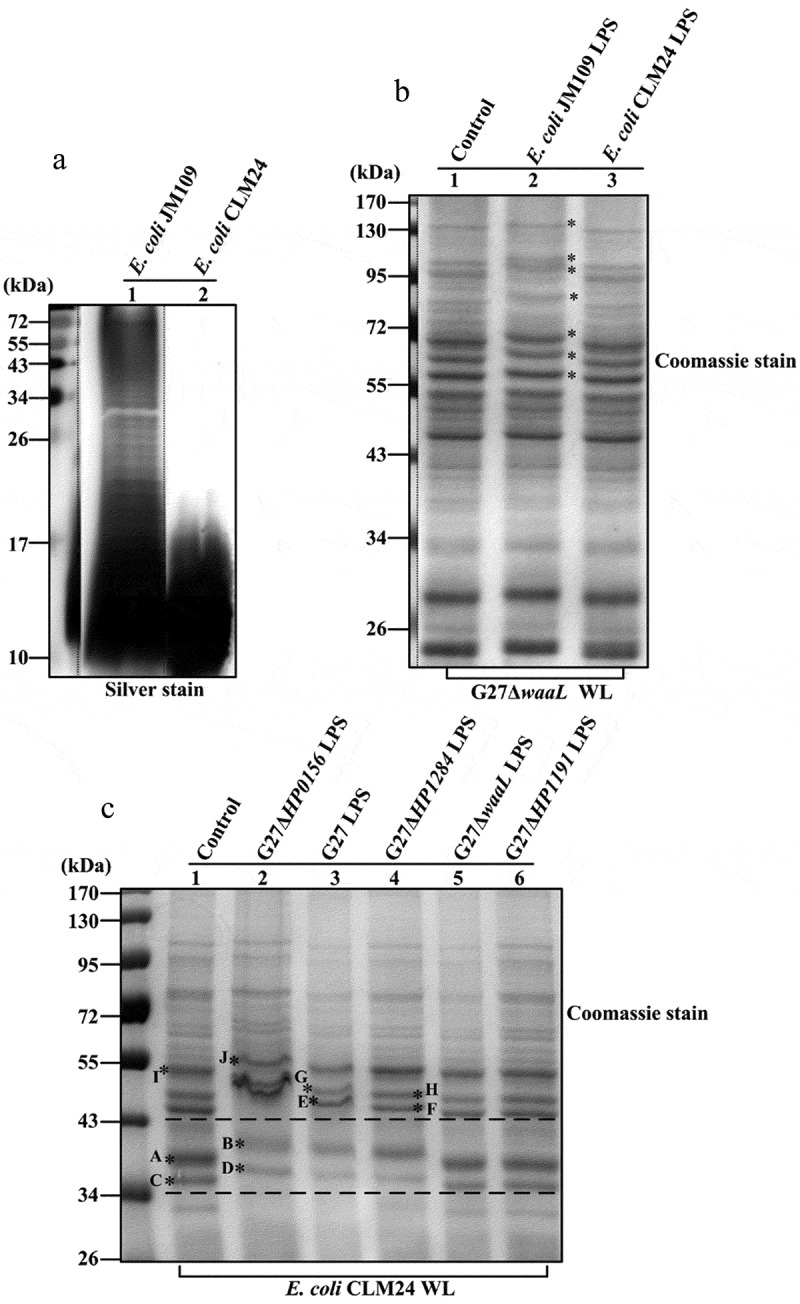
SDS-PAGE analysis of G27Δ*waaL* whole cell lysate upon the addition of different length of *E. coli* LPS, and of *E. coli* CLM24 whole cell lysate upon the addition of different length of *H. pylori* LPS. (a) Silver staining of full-length LPS extracted from *E. coli* JM109 and O-antigen truncated LPS from *E. coli* CLM24. (b) G27Δ*waaL* whole cell lysate with the addition of purified *E. coli* LPS (*indicated the clear shift in MW). (c) *E. coli* CLM24 whole cell lysate with the addition of purified *H. pylori* LPS (the bands marked with letters were excised for MS analysis). The setup total volume of the lps-protein reconstitution was 100 µl (whole cell lysate 10 µl, extracted LPS 20 µl, 10 mm tris-HCl (pH 8.0) 50 µl, 5 × SDS loading buffer 20µL). An aliquot of 10 µl of the reconstitution mixture was run on SDS-PAGE for Coomassie stain.

Since the LPS O-antigen of most *H. pylori* strains contains the LacNAc backbone chain (Gal-GlcNAc),^21^ we pondered whether this specific structure is the underlying cause of the protein electrophoretic shift in *H. pylori*. To answer this question, we extracted full-length LPS from *E. coli* strain JM109,^22,23^ and O-antigen deficient LPS from *E. coli* strain CLM24^24^ (Figure 6a), and added it to the whole cell lysate of G27Δ*waaL* for SDS-PAGE analysis (Figure 6b). Interestingly,

The addition of full-length LPS from *E. coli* JM109 induced an increase in the apparent MW of multiple protein bands in the G27Δ*waaL* whole cell lysate (compare Figure 6b lane 2 with lane 1). In contrast, no shift in apparent protein MW was observed upon the addition of O-antigen-deficient LPS from *E. coli* CLM24 (compare Figure 6b, lane 3 with lane 1). Furthermore, when *E. coli* CLM24 whole cell lysate was mixed with full-length LPS extracted from *H. pylori* G27 wild-type and G27Δ*HP0156*, an irregular electrophoretic pattern characterized by curved or diffused bands was evident on the SDS-PAGE gel (Figure 6c, lanes 2 and 3), along with an increase in apparent protein MW (compare Figure 6c, lanes 2 and 3 with lane 1). Notably, the effect is more subtle with *E. coli* LPS compared to *H. pylori* LPS, indicating that the magnitude of effect of LPS influence on protein mobility is dependent on bacterial source of the LPS. A slight increase in apparent protein MW was observed with the addition of LPS from G27Δ*HP1284*, whereas no shift in apparent protein MW was observed by the addition of O-antigen truncated LPS from mutants G27Δ*waaL* and G27Δ*HP1191*. Through protein MS analysis, the *E. coli* protein bands with a shift in apparent MW (Figure 6c, protein bands A *vs* B, C *vs* D, E *vs* F, G *vs* H, and I with J) were predominantly identified as outer membrane proteins, including OmpA, OmpC OmpF, and NmpC (Table S4).

## Discussion

Electrophoretic mobility, as assessed by SDS-PAGE, is a universal method for the determination of protein MW.^25^ Proteins with larger than predicted apparent MW induced by electrophoretic mobility on SDS-PAGE are, in most cases, indicative of post-translational modifications. In this study, using a combination of genetics, protein purification, glycosylation-specific staining, LPS structural analysis and LPS-protein reconstitution experiments, we demonstrate that RecA is not a glycoprotein dependent on the LPS biosynthetic pathway. Importantly, we demonstrate that the alteration of electrophoretic mobility or the shift in apparent MW of RecA and many other outer membrane proteins including AlpA/B is due to the presence of full-length LPS molecules rather than protein glycosylation.

RecA in *H. pylori* is highly conserved and has a predicted MW of 38 kDa (347 aa), which is consistent with SDS-PAGE observation when expressed in a shuttle plasmid in *E. coli* BL21. However, when the same plasmid was expressed in *H. pylori*, RecA migrated at a MW of 40 kDa on SDS-PAGE.^26^ This approximately 2 kDa MW shift was also observed between *H. pylori* wild-type and LPS biosynthetic gene mutants (Δ*galE* and Δ*pmi*).^10^ Consequently, *H. pylori* RecA was suggested to be glycosylated dependent on LPS biosynthetic pathway.^10^ This shift in the apparent MW of RecA on SDS-PAGE can now be satisfactorily explained by the influence of different length of LPS in *H. pylori* and *E. coli* on SDS-PAGE protein migrations, rather than protein glycosylation. As demonstrated in Figure 6, only the O-antigen containing full-length LPS (extracted either from *H. pylori* or *E. coli*) induces the increase in apparent protein MW on SDS-PAGE. The commonly used *H. pylori* reference strains including G27 and 26,695 produce full-length LPS,^17,18^ and RecA protein samples prepared from *H. pylori* wild-type strains had the presence of full-length LPS, and therefore migrated anomalously at approximately 40 kDa on SDS-PAGE, whereas RecA protein samples prepared from the O-antigen deficient *E. coli* BL21 or the O-antigen truncated *H. pylori* mutants migrated normally at 38 kDa on SDS-PAGE. It can be inferred that when *H. pylori* RecA is expressed in an *E. coli* strain producing O-antigen containing full-length LPS, the expressed RecA would migrate to ~ 40 kDa on SDS-PAGE.

Previously, when *H. pylori* cells were separated into membrane and cytoplasmic fractions, RecA from the membrane fractions of *H. pylori* wild-type and mutants exhibited a shift in apparent MW on SDS-PAGE, whereas RecA from the cytoplasmic fractions of all strains migrated similarly (38 kDa) on SDS-PAGE.^10^ As RecA was thought to be a glycoprotein at that time, it was explained that an activity which removes the modification is released during cell fractionation which does not affect the membrane-bound RecA.^10^ In this study, the negative result from the glycol-staining of RecA purified from *H. pylori* is highly suggestive that RecA is not a glycoprotein. The loss of the shift in apparent MW of RecA MW in the cytoplasmic fractions can be explained by the absence of LPS, as LPS is localized in the outer membrane, and during cell fractionation, LPS is separated into the membrane fractions.

Like the shift in apparent MW of RecA, it can be inferred the shift in apparent MW of AlpA/B, BabA/B observed between *H. pylori* wild-type and LPS mutants (Δ*wecA*, Δ*wzk*, and Δ*waaL*) in a previous study,^12^ is not indicative of protein glycosylation that is dependent on LPS biosynthetic genes, but is rather due to the influence of full-length LPS. The reported progressive two-step pattern of increased apparent MW from the cytoplasm to the inner membrane and from the inner membrane to the outer membrane observed in *H. pylori* wild type strain 26,695,^12^ can now be easily explained by the localization of LPS in the membranes. As the O-antigen ligation to the lipid A-core takes place in the inner membrane to form full-length LPS, which is then transported to the outer membrane, the full-length LPS is also present in the inner membrane, but its amount is progressively increased from the inner membrane to the outer membrane.^17,18,21^ The two-step pattern of increased apparent MW from the cytoplasm to inner membrane and then the outer membrane is simply because of the different amounts of full-length LPS, as we clearly demonstrate in the LPS-protein re-constitution experiment, where the increase in apparent protein MW is also positively correlated with the amount of full-length LPS (Figure 6).

Similarly, it can be inferred that the shift in apparent MW observed for EmaA between *A*. *actinomycetemcomitans* wild-type and LPS mutants on SDS-PAGE^16^ is very likely due to the influence of the different length of LPS in each strain. The conclusion that EmaA in *A*. *actinomycetemcomitans* is post-translationally modified by glycosylation dependent on LPS biosynthetic pathway is likely to be a misinterpretation.

The underlying mechanism of how the presence of full-length LPS induces anomalous SDS-PAGE migration of proteins remains to be answered. It has been reported that the anomalous SDS-PAGE migration of membrane proteins can be attributed to altered binding of the SDS detergent; specifically, proteins with greater SDS binding exhibit reduced migration and diffuse bands.^25^ In this context, it is possible that the carbohydrates present in the LPS *O*-antigen may facilitate the binding of more SDS to the associated proteins.

Despite the findings made in this study, there are some limitations that warrant further investigation. First, mass spectrometry analysis of the proteins, including RecA, AlpA/B, and BabA/B, purified from *H. pylori* wild-type and LPS mutants was not conducted in this study, which prevent us from providing direct evidence to support our conclusions regarding the glycosylation of these proteins. Second, the underlying mechanism by which full-length LPS modulates the migration behavior of certain proteins in SDS gels remains unresolved, as this is beyond the scope of the current study. Future studies are being planned to purify relevant proteins, including RecA and AlpA/B, and to perform protein-LPS binding experiments using surface plasmon resonance to investigate the underlying mechanisms of the interactions involved.

In summary, our study demonstrates that the presence of full-length O-antigen containing LPS interferes with protein electrophoretic mobility on SDS-PAGE. Since LPS is a unique and integral component of the outer membrane in nearly all Gram-negative bacteria, the influence of LPS on protein electrophoretic motility should be considered first in the interpretation of an observed shift in MW of protein samples prepared from Gram-negative bacteria, rather than interpreting it as potential protein glycosylation that is dependent on LPS biosynthetic pathway.

## Materials and methods

### Bacterial strains, plasmids, oligosaccharides, and culture conditions

*H. pylori* and *E. coli* strains, plasmids and oligosaccharides used in this study are listed in Table S1 & S2. *H. pylori* was routinely grown on Columbia blood agar (CBA) plates containing Columbia blood agar base (Oxoid) with 5% (v/v) horse blood and 5% (v/v) new-born calf serum supplemented with chloramphenicol (10 µg/ml) or streptomycin (10 µg/ml) where appropriate. Plates were incubated at 37°C for 24–48 h within sealed jars containing an atmosphere of N_2_: H_2:_ CO_2_ (85: 5: 10) by atmosphere replacement with the Anoxomat™ Mark II system (Mart Microbiology B.V., The Netherlands).

### Construction of G27ΔHP0156 mutant

G27Δ*HP0156* mutant was constructed using the *Xer-cise* gene deletion method.^27^ Specifically, two DNA fragments, upstream and downstream of the *HPG27_143* ORF (corresponding to *HP0156*), were amplified from G27 genomic DNA using primers HP0156-F andHP0156-BamHI-R, and HP0156-BamHI-F and HP0156-R. The two DNA fragments were joined by SOE PCR to give a 1.8-kb fragment containing the DNA regions flanking *HPG27_143* separated by a unique *Bam*HI site. This PCR product was treated with *MyTag* DNA polymerase to facilitate TA cloning into the pGEM^Ⓡ^-T Easy Vector to generate plasmid p0156-AB. The *difH* flanked *rpsL-cat* cassette was liberated from pDifWT-RC by *Bam*Hl digest and cloned into the unique *Bam*Hl site of p0156-AB to generate plasmid p0156-AB-difH-RC, which was used to transform strain G27 to generate the G27Δ*HP0156* mutant. Diagnostic PCR using chromosomal DNA was performed to confirm the loss of *HPG27_143* using primers HP0156-F andHP0156-R.

### Construction of strains expressing His_6_-tagged RecA at the ureAB locus of G27 wild-type and selected mutants

To generate the strains that express a His_6_-tagged RecA at the *ureAB* locus, recipient strains harboring *rpsL-cat* (RC) at this locus were first generated. Recipient strains were made by natural transformation of plasmid pBlueAB:RC into strains G27 wild-type, G27Δ*HP0156*, G27Δ*HP1283*, G27Δ*HP1284*, G27Δ*HP0102*, and G27Δ*waaL*, generating *ureAB* recipient strains G27*ureAB:RC*, G27Δ*HP0156ureAB:RC*, G27Δ*HPI283ureAB:RC*, G27Δ*HP1284 ureAB:R*C，G27Δ*HP0102ureAB:RC*, and G27Δ*waaLureAB:RC*, respectively.

The DNA fragment “*his*_*6*_*-recA-ha*” was amplified from 26,695 genomic DNA using primers RecA-His_6_-tag F and RecA HA epitope R to amplify the *recA* ORF (HP0153) and introduce sequences that encoded a *N*-terminal His_6_ tag and a C-terminal HA tag. The “*his*_*6*_*-recA-ha*” PCR product was digested with *Ecor*RI and *Bgl*II and cloned into the corresponding restriction sites of pBlueAB to give pBlueAB-(*his*_*6*_*-recA-ha*). This plasmid was used to transform the *ureAB* recipient strains constructed above to generate strains expressing a His_6_-tagged RecA: G27Δ*HP1283ureAB:his*_*6*_*-recA-ha*, G27Δ*HP0156ureAB::his*_*6*_*-recA-ha*, G27Δ*HP1284 ureAB:his*_*6*_*-recA-ha*, G27Δ*HP0102ureAB:his*_*6*_*-recA-ha*, and G27Δ*waaLureAB:his*_*6*_*-recA-ha*.

### Purification of his-tagged RecA and glycol-staining

His_6_-tagged RecA was purified from *H. pylori* strains using the HisPur™ Ni-NTA Resin under native conditions according to the manufacturer’s instructions. Briefly, bacterial cells (expressing His_6_-tagged RecA) grown on 10 blood agar plates for 24 h were harvested into 20 mL of cold PBS buffer. After thorough suspension, the bacterial cells were centrifuged down at 4,000 rpm for 10 min at 4°C. The bacterial cells were resuspended with 5 mL lysis buffer (20 mm NaH_2_PO_4_, 500 mm NaCl, 1% Triton, pH 7.2). The bacterial suspension was then incubated in an ice water bath for 1 h. The unlysed bacterial cells and cell debris were removed by centrifugation at 12,000 rpm for 10 min at 4°C. The resulting supernatant was transferred to a 14 mL conical tube and 5 mL of buffer A (20 mm NaH_2_PO_4_, 500 mm NaCl, pH 7.2) was added to the solution to dilute the Triton X-100 concentration down to 0.5%. The suspension was then mixed with 250 μL of equilibrated Ni-NTA Resin, and the tube was rotated for 30 min at 4°C. Finally, the resin with the bound His_6_-tagged RecA was eluted with different concentrations of imidazole (25 mm to 250 mm) in buffer B (20 mm NaH_2_PO_4_ 500 mm NaCl, 0.5% Triton, pH 7.2). The eluted solutions were loaded on SDS-PAGE for Coomassie staining and glycol-staining using the GlycoProfile III Fluorescent Glycoprotein Detection Kit (Sigma-Aldrich, Inc.).

### LPS extraction or purification for Western blot and structural analysis

Crude preparation of LPS from *H. pylori* wild-type and mutants was used for silver staining or Western blot.^17^ For uptitration of LPS into G27Δ*waaL* whole cell lysate, LPS was extracted using an LPS Extraction Kit (iNtRON Biotechnology, Inc.). For G27Δ*HP0156* LPS structural analysis, LPS from G27Δ*HP0156* was extracted on a large scale using the hot phenol-water method and then structurally analyzed by mass spectrometry and NMR spectroscopy as previously described.^17^

### Preparation of whole cell lysates and outer membrane fractions

Bacterial cells grown on blood agar plates for 24 h were harvested directly into PBS and the optical density was standardized to OD_600_ = 3. The standardized sample was heated at 95°C for 10 min, sonicated for 10 s and then stored at −20°C until it was run on SDS-PAGE for Coomassie stain and Western blot.

Outer membrane fractions were extracted by sarcosine cell fractionation according to known methods^28^ with modifications. In brief, bacterial cells collected from three blood agar plates (1 day growth) were suspended in 10 ml of cold 20 mm Tris (pH 7.5) buffer. The bacterial cells were sonicated on ice, and the unbroken cells and cellular debris were then removed by centrifugation at 12,000 *g* for 10 min at 4°C. The supernatant was transferred to a new tube and the total membranes were collected by ultra-speed centrifugation at 27,500 rpm (129,676 *g*) for 45 min at 4°C. The total membranes (the pellet) were re-suspended in 20 mm Tris (pH 7.5) buffer containing 2.0% sodium lauryl sarcosine and incubated for 30 min at room temperature. This solution was then subjected to sonication to achieve complete re-suspension of the total membrane fraction and then the separation of outer membranes from inner membranes was achieved by ultra-speed centrifugation at 27,500 rpm (129,676 *g*) for 45 min at 4°C. The outer membrane fractions (the pellet) were re-suspended in 100 μL of 20 mm Tris (pH 7.5,) buffer containing 2% SDS (w/v), and stored at −20°C until analysis by Coomassie stain and Western blot.

### Identification of proteins by mass spectrometry

The protein bands of interest were excised from Coomassie stained SDS PAGE gels and sent to Proteomics International Laboratories Ltd for identity analysis by electrospray (LC-MS/MS) mass spectrometry.

## Supplementary Material

Supplemental Material

## Data Availability

The authors confirm that the data supporting the findings of this study are available within the article and its supplementary materials. Of note, this study is an extension of a portion of my PhD thesis: Li, H. (2014). Lipopolysaccharide biosynthesis and protein glycosylation in Helicobacter pylori (PhD thesis, The University of Western Australia). Link: https://api.research-repository.uwa.edu.au/ws/portalfiles/portal/9174632/Li_Hong_2014.pdf.
