# Supplementary material for: Reinvestigation into the role of lipopolysaccharide Glycosyltransferases in Helicobacter pylori protein glycosylation
Source: Gut Microbes. 2025 Jan 20;17(1):2455513. doi: 10.1080/19490976.2025.2455513 (PMC12931685; doi:10.1080/19490976.2025.2455513)
Supplement: Supplemental Material [file KGMI_A_2455513_SM0649.zip › Table S4 MS protein.docx]

Table S4. MS analysis of the paired protein bands excised from the SDS-PAGE of *E. coli* CLM24 whole lysate (Fig 6).

| **ID** | **Protein hits** (Seq. Ref) | **ID** | **Proteins hits** (Seq. Ref) |
| --- | --- | --- | --- |
| **A** | OmpC (I4U3N9) | **B** | OmpC (I4U3N9) |
|  | Putative Omp (B6I1B6) |  | Putative Omp (B6I1B6) |
|  | OmpF (G0D5E3) |  | Outer membrane protein F (G0D5E3) |
|  | Outer membrane porin protein NmpC (L4UI35) |  | Outer membrane porin protein NmpC (L4UI35) |
|  | GapA Glyceraldehyde-3-phosphate dehydrogenase A (P0A9B2) |  |  |
| **C** | OmpA (P0A911) | **D** | OmpA (P0A911) |
|  | CysK Cysteine synthase A (P0ABK5) |  |  |
| **E** | EF-Tu (P0CE48) | **F** | EF-Tu (P0CE48) |
| **G** | Enolase (I2WWD7) | **H** | Enolase (I2WWD7) |
|  | AceA Isocitrate lyase (P0A9G7) |  | AceA Isocitrate lyase (P0A9G7) |
| **I** | GadA (Glutamate decarboxylase) (A5YKF4) | **J** | GadA (Glutamate decarboxylase) (A5YKF4) |
|  | GadA (Glutamate decarboxylase alpha) (G0D5U1) |  | GadA (Glutamate decarboxylase alpha) (G0D5U1) |
|  | Glutamate decarboxylase (E1J949) |  |  |
