# Supplementary material for: Reinvestigation into the role of lipopolysaccharide Glycosyltransferases in Helicobacter pylori protein glycosylation
Source: Gut Microbes. 2025 Jan 20;17(1):2455513. doi: 10.1080/19490976.2025.2455513 (PMC12931685; doi:10.1080/19490976.2025.2455513)
Supplement: Supplemental Material [file KGMI_A_2455513_SM0649.zip › Table S1 Bacterial Strains and plasmids.docx]

**Table S1.** Bacterial strains and plasmids used in this study

| **Strain or plasmid name** | **Description** | **LPS structure** |
| --- | --- | --- |
| ***H. pylori* strains** |  |  |
| G27 | Wild-type strain | Full-length LPS |
| G27Δ*HP0156* | Deletion of *HPG27_143* | Full-length LPS |
| G27Δ*HP1105* | Deletion of *HPG27_1046* | Lack Lewis antigen |
| G27Δ*HP0826* | Deletion of *HPG27_785* | Lack Lewis antigen |
| G27Δ*HP1578* | Deletion of *HPG27_1515* | Lack Lewis antigen |
| G27Δ*HP1283* | Deletion of *HPG27_1235* | Lack Heptan-Lewis antigen |
| G27Δ*HP0159* | Deletion of *HPG27_146* | Lack Glucan-Heptan-Lewis antigen |
| G27Δ*HP0479* | Deletion of *HPG27_437* | Lack Trio Hep-Glucan-Heptan-Lewis antigen |
| G27Δ*HP0102* | Deletion of *HPG27_94* | Lack Trio Fuc-Hep-Glucan-Heptan-Lewis antigen |
| G27Δ*wecA* | Deletion of *HPG27_1518* | Lack whole O-antigen |
| G27Δ*wzk* | Deletion of *HPG27_1153* | Lack whole O-antigen |
| G27Δ*waaL* | Deletion of *HPG27_389* | Lack whole O-antigen |
| G27Δ*HP0805* | Deletion of *HPG27_761* | Lack branched disaccharide only |
| G27Δ*HP1416* | Deletion of *HPG27_1339* | Lack branched Glc only |
| G27Δ*HP1284* | Deletion of Deletion of *HPG27_1236* | Lack Hep III and the attached disaccharide |
| G27Δ*HP1191* | Deletion of *HPG27_1136* | Deep truncation from Hep II residue |
| G27*ureAB::RC* | *rpsL-cat* cassette inserted at the *ureAB* locus |  |
| G27*ureAB::his*_6_*-recA-ha* | *his_6_-recA-ha* expressed at the *ureAB* locus |  |
| G27Δ*HP0156ureAB::RC* | *rpsL-cat* cassette inserted at the *ureAB* locus |  |
| G27Δ*HP0156ureAB::his_6_-recA-ha* | *his_6_-recA-ha* expressed at the *ureAB* locus |  |
| G27Δ*HPI283ureAB::RC* | *rpsL-cat* cassette inserted at the *ureAB* locus |  |
| G27Δ*HP1283ureAB::his_6_-recA-ha* | *his_6_-recA-ha* expressed at the *ureAB* locus |  |
| G27Δ*HP1284ureAB::R*C | *rpsL-cat* cassette inserted at the *ureAB* locus |  |
| G27Δ*HP1284ureAB::his_6_-recA-ha* | *his_6_-recA-ha* expressed at the *ureAB* locus |  |
| G27Δ*HP0102ureAB::RC* | *rpsL-cat* cassette inserted at the *ureAB* locus |  |
| G27Δ*HP0102ureAB::his_6_-recA-ha* | *his_6_-recA-ha* expressed at the *ureAB* locus |  |
| G27Δ*waaLureAB::RC* | *rpsL-cat* cassette inserted at the *ureAB* locus |  |
| G27Δ*waaLureAB::his_6_-recA-ha* | *his_6_-recA-ha* expressed at the *ureAB* locus |  |
| ***E. coli* strains** |  |  |
| JM109 | Wild-type strain | Full-length LPS |
| CLM24 | *E. coli* W3110 by the deletion *waaL* | Lack O-antigen |
| **Plasmids** |  |  |
| pDifWT-RC | *rpsL-cat* cassette flanked by *H. pylori* *difH* sequence |  |
| p0156-AB | pGEM^®^-T Easy vector containing sequences flanking *HPG27_143*, separated by a *Bam*HI restriction site |  |
| p0156-AB-difH-RC | Derivative of p0156-AB, with *difH-rpsL-cat-difH* inserted between *HPG27_143* flanking sequences at the *Bam*HI site |  |
| pBlueAB | Derivative of pBlueScript SK containing *ureA* and *ureB* sequences from strain 26695, separated by *Ecor*RI and *Bgl*II restriction sites |  |
| pBlueAB::RC | Derivative of pBlueAB with *rpsL-cat* cassette inserted between *ureA* and *ureB* |  |
| pBlueAB-(his_6_-recA-ha) | Derivative of pBlueAB containing *recA* with a *N*-terminal His_6_ tag and C-terminal HA tag |  |
