# Supplementary material for: Reinvestigation into the role of lipopolysaccharide Glycosyltransferases in Helicobacter pylori protein glycosylation
Source: Gut Microbes. 2025 Jan 20;17(1):2455513. doi: 10.1080/19490976.2025.2455513 (PMC12931685; doi:10.1080/19490976.2025.2455513)
Supplement: Supplemental Material [file KGMI_A_2455513_SM0649.zip › Table S3 LPS titration assay setup.docx]

**Table S3.** The setup for LPS-protein reconstitution assay.

| Setup | LPS uptitration samples | | | | | | |
| --- | --- | --- | --- | --- | --- | --- | --- |
|  |  | | | | | | |
| Extracted LPS^*^ (µL) | 0 | 2 | 4 | 8 | 16 | 32 | 64 |
| 10 mM Tris-HCl (pH 8.0) (µL) | 70 | 68 | 64 | 62 | 54 | 38 | 6 |
| 5× SDS loading buffer (µL) | 20 | 20 | 20 | 20 | 20 | 20 | 20 |
| G27Δ*waaL* whole cell lysate (µL) | 10 | 10 | 10 | 10 | 10 | 10 | 10 |
| Total volume (µL)^#^ | 100 | 100 | 100 | 100 | 100 | 100 | 100 |

^*^LPS was extracted using the LPS Extraction Kit, and the concentration of extracted LPS was estimated to be 1.8 µg/µL according to the Kit protocol; ^#^ 10 µL of each titration sample was run on SDS-PAGE for Coomassie stain and Western blot.
