# Supplementary material for: Reinvestigation into the role of lipopolysaccharide Glycosyltransferases in Helicobacter pylori protein glycosylation
Source: Gut Microbes. 2025 Jan 20;17(1):2455513. doi: 10.1080/19490976.2025.2455513 (PMC12931685; doi:10.1080/19490976.2025.2455513)
Supplement: Supplemental Material [file KGMI_A_2455513_SM0649.zip › Table S2 Primers used in this study.docx]

**Table S2.** Oligonucleotides used in this study

| **Primer name** | **Sequence (5’-->3’)** | **Function of PCR product** |
| --- | --- | --- |
| HP0156-F | GAGCAGCGAATTGGTGGATG | Construction of p0156-AB-difH-RC |
| HP0156-BamHI R | CTAAATGCTGCATGCTTAGATCTTGCCATTTCCTTAAATCTAATCTC |  |
| HP0156-BamHI F | GCAAGATCTAAGCATGCAGCATTTAGTCTTAATCG |  |
| HP0156-R | CCTCAAAGGGATTTGCAAGCAC |  |
| RecA-His_6_-tag F | GACGACGAATTCATGCATCATCACCATCACCACGCAATAGATGAAGACAAACAAAAAGC | Tagging RecA |
| RecA-HA-epitope R | GTCGTCAGATCTTTATTCTTTAGGAAAAATTTCAAATCTTTCAAAGCTTTCCATTTCTTCTAAAGGCTCATCC |  |
